# A Neurocomputational Model of the Mismatch Negativity

**DOI:** 10.1371/journal.pcbi.1003288

**Published:** 2013-11-07

**Authors:** Falk Lieder, Klaas E. Stephan, Jean Daunizeau, Marta I. Garrido, Karl J. Friston

**Affiliations:** 1 Translational Neuromodeling Unit (TNU), Institute of Biomedical Engineering, University of Zurich & ETH Zurich, Zurich, Switzerland; 2 Laboratory for Social and Neuronal Systems Research, Dept. of Economics, University of Zurich, Zurich, Switzerland; 3 Helen Wills Neuroscience Institute, University of California at Berkeley, Berkeley, California, United States of America; 4 Wellcome Trust Centre for Neuroimaging, Institute of Neurology, University College London, London, United Kingdom; 5 Brain and Spine Institute (ICM), Paris, France; 6 Queensland Brain Institute, The University of Queensland, St Lucia, Australia; Indiana University, United States of America

## Abstract

The mismatch negativity (MMN) is an event related potential evoked by violations of regularity. Here, we present a model of the underlying neuronal dynamics based upon the idea that auditory cortex continuously updates a generative model to predict its sensory inputs. The MMN is then modelled as the superposition of the electric fields evoked by neuronal activity reporting prediction errors. The process by which auditory cortex generates predictions and resolves prediction errors was simulated using generalised (Bayesian) filtering – a biologically plausible scheme for probabilistic inference on the hidden states of hierarchical dynamical models. The resulting scheme generates realistic MMN waveforms, explains the qualitative effects of deviant probability and magnitude on the MMN – in terms of latency and amplitude – and makes quantitative predictions about the interactions between deviant probability and magnitude. This work advances a formal understanding of the MMN and – more generally – illustrates the potential for developing computationally informed dynamic causal models of empirical electromagnetic responses.

## Author Summary

Computational neuroimaging enables quantitative inferences from non-invasive measures of brain activity on the underlying mechanisms. Ultimately, we would like to understand these mechanisms not only in terms of physiology but also in terms of computation. So far, this has not been addressed by mathematical models of neuroimaging data (e.g., dynamic causal models), which have rather focused on ever more detailed inferences about physiology. Here we present the first instance of a dynamic causal model that explains electrophysiological data in terms of computation rather than physiology. Concretely, we predict the mismatch negativity – an event-related potential elicited by regularity violation – from the dynamics of perceptual inference as prescribed by the free energy principle. The resulting model explains the waveform of the mismatch negativity and some of its phenomenological properties at a level of precision that has not been attempted before. This highlights the potential of neurocomputational dynamic causal models to enable inferences from neuroimaging data on neurocomputational mechanisms.

## Introduction

Recent advances in computational neuroimaging [1] have enabled inferences about the neurophysiological mechanisms that generate non-invasive measures of task or stimulus-evoked neuronal responses; as measured by functional magnetic resonance imaging (fMRI) or electroencephalography (EEG). One such approach is dynamic causal modelling [2] that tries to explain EEG data in terms of synaptic coupling within a network of interacting neuronal populations or sources. However, this description is at the level of physiological processes that do not have a direct interpretation in terms of information processing. Cognitive scientists have been using formal models of cognitive processes to infer on information processing from behaviour for decades [3], but it has remained largely unclear how such inferences should be informed by neurophysiological data. We argue that one may overcome the limitations of both approaches by integrating normative models of information processing (e.g., [4], [5]) with physiologically grounded models of neuroimaging data [4], [5]. This approach may produce computationally informed neuronal models – or neurocomputational models – enabling one to test hypotheses about how the brain processes information to generate adaptive behaviour. Here, we provide a proof-of-concept for this approach by jointly modelling a cognitive process – perceptual inference – and the event related potential (ERP) that it may generate – the mismatch negativity (MMN). Specifically, we ask whether the MMN can be modelled by a neuronal system performing perceptual inference, as prescribed by predictive coding [4], [5].

The MMN is an event-related potential that is evoked by the violation of a regular stream of sensory events. By convention, the MMN is estimated by subtracting the ERP elicited by *standards*, i.e. events that established the regularity, from the ERP elicited by *deviants*, i.e. events violating this regularity. Depending on the specific type of regularity, the MMN is usually expressed most strongly at fronto-central electrodes, with a peak latency between 100 and 250 milliseconds after deviant onset [1]. More precisely, the MMN has been shown to depend upon deviant probability and magnitude. Deviant probability is the relative frequency of tones that violate an established regularity. In studies of the MMN evoked by changes in sound frequency, deviance magnitude is the (proportional) difference between the deviant frequency and the standard frequency. The effects of these factors are usually summarized in terms of changes in the MMN peak amplitude and its latency (see Table 1). While increasing the deviance magnitude makes the MMN peak earlier and with a larger amplitude [4], [6], [7], decreasing deviant probability only increases the MMN peak amplitude [8] but does not change its latency [9].

**Table 1:** Overview of the Phenomenological Properties of the MMN.

Effect of ↓ on →	|MMN Amplitude|	MMN Latency
Higher Deviance Magnitude	**↗** [9]	** 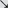 ** [11]
Lower Deviant Probability	**↗** [9]	no effect [9]

The question as to which neurophysiological mechanisms generate the MMN remains controversial (cf. [10] vs. [11]), even though this issue has been addressed by a large number of studies over the last thirty years [12]. One reason for an enduring controversy could be that the MMN's latency and amplitude contain insufficient information to disambiguate between competing hypotheses (but see [13]). While the MMN is the sum of overlapping subcomponents that are generated in temporal and frontal brain areas [12], [14] – and are differentially affected by experimental manipulations [15] – it is a continuous function of time. This means that the underlying ERP waveforms may contain valuable information about MMN subcomponents, the physiological mechanisms that generate them and, critically, their functional correlates (see e.g. [16]). Predictive coding offers a unique and unified explanation of the MMN's neurophysiological features. In brief, predictive coding is a computational mechanism that formally links perception and learning processes to neural activity and synaptic plasticity, respectively [17]. More precisely, event-related electrophysiological responses are thought to arise from the brain's attempt to minimize prediction errors (i.e. differences between actual and predicted sensory input) through hierarchical Bayesian inference. In this context, the MMN simply reflects neuronal activity reporting these prediction errors in hierarchically organized network of auditory cortical sources. If this is true, then the rise and fall of the MMN may reflect the appearance of a discrepancy between sensory input and top-down predictions – and its resolution through perceptual inference. These ideas have been used to interpret the results of experimental studies of the MMN [8], [18] and computational treatments of trial-wise changes in amplitude [6]. However, no attempt has been made to quantitatively relate predictive coding models to empirical MMN waveforms. Here, we extend these efforts by explicitly modelling the physiological mechanisms underlying the MMN in terms of a computational mechanism: predictive coding. In other words, our model is both an extension to dynamic causal models of observed electrophysiological responses [18], [19] to information processing, and a neurophysiological view on meta-Bayesian approaches to cognitive process [15]. We establish the face validity of this neurocomputational model in terms of its ability to explain the observed MMN and its dependence on deviant frequency and deviance magnitude.

This paper comprises two sections. In the first section, we summarize mathematical models of predictive coding (as derived from the free energy principle), and describe the particular perceptual model that we assume the brain uses in the context of a predictable stream of auditory stimuli. The resulting scheme provides a model of neuronal responses in auditory oddball paradigms. In line with the DCM framework, we then augment this model with a mapping from (hidden) neuronal dynamics to (observed) scalp electrophysiological data. In the second section, we use empirical ERPs acquired during an oddball paradigm to tune the parameters of the observation model. Equipped with these parameters, we then simulate MMN waveforms under different levels of deviant probability and deviance magnitude – and compare the resulting latency and amplitude changes with findings reported in the literature. This serves to provide a proof of principle that dynamic causal models can have a computational form – and establish the face validity of predictive coding theories brain function.

## Models

**Figure 1:**
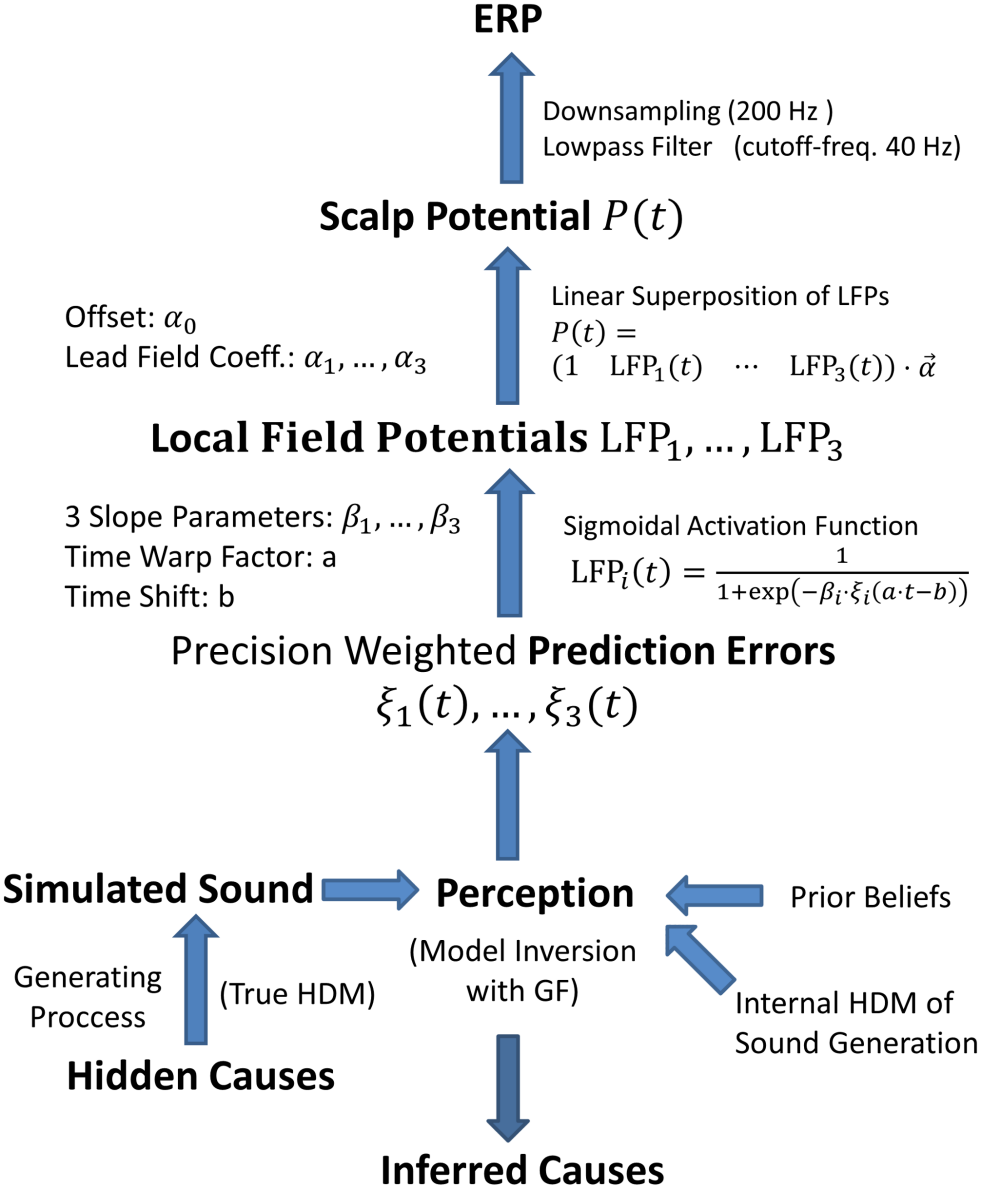
Flow Chart of MMN simulations. Sensory input was generated from a Hierarchical Dynamic Model (true HDM) for a standard or deviant stimulus. This stimulus was produced by inputs controlling the temporal evolution of loudness and frequency (hidden causes). We simulated perception with the inversion of the internal model (internal HDM) of a subject – who anticipates the standard event with a certain degree of confidence (prior beliefs) – with Generalised Filtering (GF). This produces a simulated trajectory of the prediction errors that are minimised during perceptual inference. These prediction errors were weighted by their precisions and used to predict event related potentials. Model parameters are listed on the left and model equations are provided on the right. To map prediction errors to empirical responses, they were shifted and scaled so that the simulated stimulus duration was 70 ms. A sigmoid function was applied to model nonlinearities in the relationship between prediction error and equivalent current dipole activity. Third, the scalp potential at the simulated electrode location was modelled as a linear superposition of the ensuing local field potentials. Finally, the simulated EEG data was down-sampled and sheltered.

To simulate the MMN under the predictive coding hypothesis, we simulated the processing of standard and deviant stimuli using established Bayesian filtering (or predictive coding) – under a hierarchical dynamic model of repeated stimuli. This generates time-continuous trajectories, encoding beliefs (posterior expectations and predictions) and prediction errors. These prediction errors were then used to explain the MMN, via a forward model of the mapping between neuronal representations of prediction error and observed scalp potentials. In this section, we describe the steps entailed by this sort of modelling. See Figure 1 for an overview.

### Predictive coding and hierarchical dynamic models

Perception estimates the causes (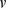) of the sensory inputs (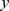) that the brain receives. In other words, to recognise causal structure in the world, the brain has to invert the process by which its sensory consequences are generated from causes in the environment. This view of perception as unconscious inference was introduced by Helmholtz [2] in the 19^th^ century. More recently, it has been formalized as the inversion of a generative model 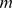 of sensory inputs 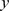 [20]. In the language of probability theory, this means that the percept corresponds to the posterior belief 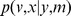 about the putative causes 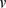 of sensory input 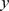 and any hidden states 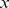 that mediate their effect. This means that any perceptual experience depends on the model 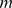 of how sensory input is generated. To capture the rich structure of natural sounds, the model 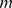 has to be dynamic, hierarchical, and nonlinear. Hierarchical dynamic models (HDMs) [21] accommodate these attributes and can be used to model sounds as complex as birdsong [22].

HDMs generate time-continuous data 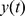 as noisy observations of a nonlinear transformation 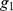 of hidden states 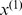 and hidden causes 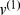:
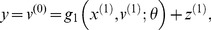
(1)where the temporal evolution of hidden states 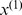 is given by the differential equation:
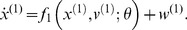
(2)This equation models the change in 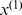 as a nonlinear function 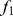 of the hidden states 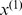 and hidden causes 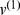 plus state noise 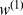. The hidden causes 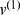 of the change in 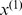 are modelled as the outputs of a hidden process at the second level. This second process is modelled in the same way as the hidden process at the first level, but with new nonlinear functions 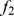 and 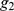:
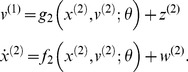
(3)As in the first level, the hidden dynamics of the second level are driven by hidden causes 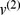 that are modelled as the output of a hidden process at the next higher level, and so forth. This composition can be repeated as often as necessary to model the system under consideration – up to the last level, whose input is usually modelled as a known function of time plus noise:
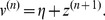
(4)The (Bayesian) inversion of HDMs is a difficult issue, which calls for appropriate approximation schemes. To explain how the brain is nevertheless able to recognise the causes of natural sounds, we assume that it performs *approximate Bayesian inference* by minimising variational free energy [23]. More generally, the free-energy principle is a mathematical framework for modelling how organisms perceive, learn, and make decisions in a parsimonious and biologically plausible fashion. In brief, it assumes that biological systems like the brain solve complex inference problems by adopting a parametric approximation 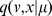 to a posterior belief over hidden causes and states 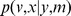. It then optimises this approximation by minimizing the variational free-energy:
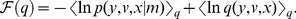
(5)One can think of this free-energy as an information theoretic measure of the discrepancy between the brain's approximate belief about the causes of sensory input and the true posterior density. According to the free-energy principle, cognitive processes and their neurophysiological mechanisms serve to minimize free-energy [24] – generally by a gradient descent with respect to the sufficient statistics 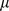 of the brain's approximate posterior 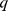 [5]:
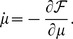
(6)This idea that the brain implements perceptual inference by free-energy minimization is supported by a substantial amount of anatomical, physiological, and neuroimaging evidence [4]. Algorithms that invert HDMs by minimizing free-energy, such as dynamic expectation maximization [25], [26] and generalized filtering (GF) [4], [5], [23], [27], [28], are therefore attractive candidates for simulating and understanding perceptual inference in the brain.

Importantly, algorithmic implementations of this gradient descent are formally equivalent to predictive coding schemes. In brief, representations (sufficient statistics encoding approximate posterior expectations) generate top-down predictions to produce prediction errors. These prediction errors are then passed up the hierarchy in the reverse direction, to update posterior expectations. This ensures an accurate prediction of sensory input and all its intermediate representations. This hierarchal message passing can be expressed mathematically as a gradient descent on the (sum of squared) prediction errors 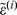 which are weighted by their precisions (inverse variances) 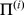:
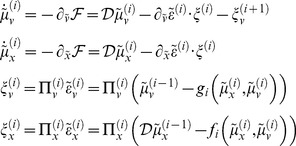
(6b)where 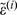 are prediction errors and 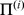 are their precisions (inverse variances). Here and below, the ∼ notation denotes generalised variables (state, velocity, acceleration and so on). The first pair of equalities just says that posterior expectations about hidden causes and states 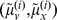 change according to a mixture of prior prediction– the first term – and an update term in the direction of the gradient of (precision-weighted) prediction error. The second pair of equations expresses precision weighted prediction error 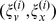 as the difference between posterior expectations about hidden causes and (the changes in) hidden states and their predicted values (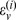,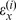), weighed by their precisions 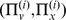. The predictions are nonlinear functions of expectations at each level of the hierarchy and the level above. In what follows, this predictive coding formulation will serve to simulate perceptual recognition. We will then use prediction errors as a proxy for neuronal activity producing ERPs. To simulate neuronal processing using Equation 6, we need to specify the form of the functions 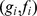 that constitute the generative model:

### The generative (auditory) model

**Figure 2:**
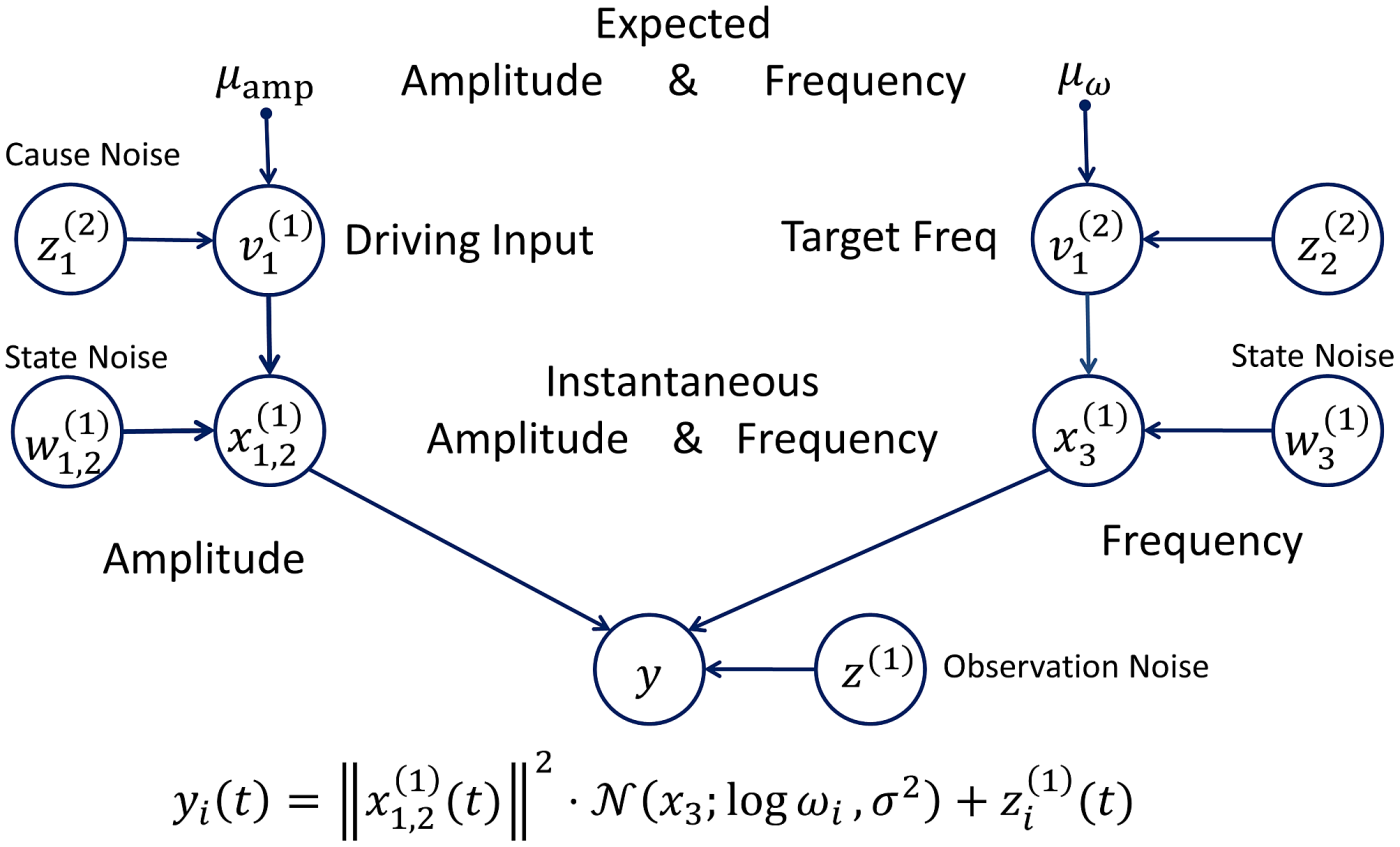
Hierarchical dynamical model of stimulus generation. This figure shows the form of the hierarchical dynamic model used to generate and subsequently recognise stimuli. The sensory input (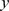)is modelled as a vector of amplitude-modulated frequency channels 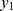 whose values are nonlinear functions of the hidden states 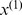 plus observation noise. The hidden states represent the instantaneous loudness (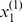 and 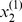) and frequency (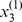). The temporal evolution of these hidden states is determined by a nonlinear random differential equation that is driven by hidden causes (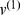). The mean of the subject's belief (posterior expectation) about hidden causes and states is denoted by 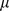. The tilde denotes variables in generalised coordinates of motion.

To model auditory cortical responses, we assume that cortical sources embody a hierarchical model of repeated stimuli. In other words, the hierarchical structure of the auditory cortex recapitulates the hierarchical structure of sound generation (cf. [25]). This hierarchical structure was modelled using the HDM illustrated in Figure 2. Note that this model was used to both generate stimuli and simulate predictive coding – assuming the brain is using the same model. The model's sensory prediction 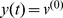 took the form of a vector of loudness modulated frequency channels (spectrogram) at the lowest level. The level above models temporal fluctuations in instantaneous loudness (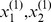) and frequency (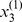). The hidden causes 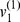 and 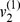 of these fluctuations are produced by the highest level. These three levels of representation can be mapped onto three hierarchically organized areas of auditory cortex: primary auditory cortex (A1), lateral Heschl's gyrus, and inferior frontal gyrus.

A1 and lateral Heschl's gyrus contain neuronal units encoding posterior expectations and prediction errors, respectively. The activity of the expectation units encodes the time course of 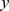 for A1 and expectations about hidden states 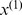 for Heschl's gyrus. Error units encode prediction error, i.e. the difference between posterior expectations and top-down predictions. Top-down connections therefore convey predictions, whereas bottom-up connections convey prediction errors. The hidden causes are the expectations of 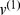, providing top-down projections from units in inferior frontal gyrus.

Our model respects the tonotopic organization of primary auditory cortex (see e.g. [26]) by considering 50 frequency channels 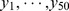. It also captures the fact that, while most neurons in A1 have a preferred frequency, their response also increases with loudness [29]–[31]. Specifically, we assume that the activity 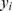 of neurons selective for frequency 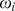 is given by:
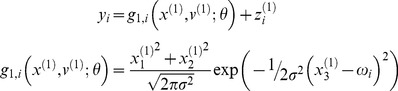
(7)We can rewrite this equation in terms of the loudness 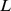 and a tuning function 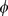 that measures how close the log-frequency 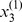 is to the neuron's preferred log-frequency 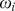:
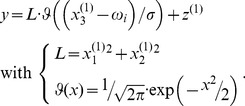
(8)This is our (perceptual) model of how the frequency and loudness is encoded by frequency-selective neurons in primary auditory cortex. We use it to simulate the activity of A1 neurons.

Note that a neuronal representation of 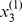 depends only on frequency. In the brain, frequency representations that are invariant to the sound level (and other sound attributes) are found in higher auditory areas; for instance in marmoset auditory cortex [32]. Neuroimaging in humans suggests that periodicity is represented in lateral Heschl's gyrus and planum temporale [33], and LFP recordings from patients again implicate lateral Heschl's gyrus [34]. We therefore assume that 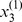 is represented in lateral Heschl's gyrus. The dynamics of the instantaneous frequency 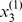 is given by
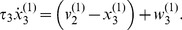
(9)This equation says that the instantaneous frequency converges towards the current target frequency 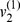 at a rate of 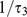. In the context of communication, one can think of the target frequency as the frequency that an agent intends to generate, where the instantaneous frequency 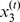 is the frequency that is currently being produced. The motivation for this is that deviations from the target frequency will be corrected dynamically over time. The agent's belief about 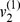 reflects its expectation about the frequency of the perceived tone and its subjective certainty or confidence about that expectation. Therefore, the effect of the deviant probability – in an oddball paradigm – can be modelled via the precision of this prior belief.

The temporal evolution of the hidden states 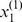 and 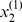 (encoding loudness) was modelled with the following linear dynamical system:
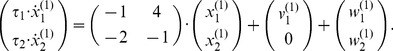
(10)In this equation the first hidden cause 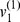 drives the drives the dynamics of hidden states, which spiral (decay) towards zero in its absence. Finally, our model makes the realistic assumption that the stochastic perturbations are smooth functions of time. This is achieved by assuming that the derivatives of the stochastic perturbations are drawn from a multivariate Gaussian with zero mean:
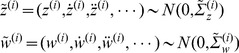
(11)The parameters of this model were chosen according to the biological and psychological considerations explained in Supplementary Text S1.

### Modelling perception

Having posited the relevant part of the generative model embodied by auditory cortex, one can now proceed to its inversion by the Bayesian generalized filtering scheme described in Equation 6. This is the focus of the next section, which recapitulates how auditory cortex might perceive sound frequency and amplitude using predictive coding mechanisms, given the above hierarchal dynamic model.

#### Perception as model inversion by generalised filtering

Generalised filtering or predictive coding (Equation 6) provides a process model of how auditory cortex might invert the model above, yielding posterior estimates of (hidden) sensory causes 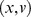 from their noisy consequences 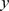. Generalised filtering (GF) [35], [36] is a computationally efficient scheme for variational Bayesian inference on hierarchical dynamical systems. This makes it a likely candidate mechanism for typical recognition problems that the brain solves when perceiving stimulus sequences.

Generalised filtering effectively updates posterior expectations by accumulating evidence over time. Since it is well known that neuronal population activity integrates inputs in a similar way [37], we take generalised filtering as a model of neuronal evidence accumulation or predictive coding (cf. [26]). The neuronal implementation of this filtering is based on the anatomy of cortical microcircuits and rests on the interaction between error units and expectation units implicit in Equation 6. Irrespective of the neuronal details of the implementation, prediction error units are likely to play a key role, because (precision weighted) prediction errors determine the free-energy gradients that update posterior beliefs about hidden states. It has been argued that prediction error units correspond to pyramidal neurons in the superficial layers of cortex [38]. Since these neurons are the primary source of local field potentials (LFP) and EEG signals, the time course of prediction errors can – in principle – be used to model event related potentials such as the MMN.

#### Modelling expectations and perception in MMN experiments

To simulate how MMN features (such as amplitude and latency) depend upon deviant probability and magnitude, we assumed that the subject has heard a sequence of standard stimuli (presented at regular intervals) and therefore expects the next stimulus to be a standard. Under Gaussian assumptions this prior belief is fully characterized by its mean – the expected attributes of the anticipated stimulus – and precision (inverse variance). The precision determines the subject's certainty about the sound it expects to hear; in other words, the subjective probability that the stimulus will have the attributes of a standard. This means one can use the expected precision to model the effect of the deviant probability in oddball paradigms – as well as the effects of the number of preceding standards. The effect of deviance magnitude was simulated by varying the difference between the expected and observed frequency. Sensory inputs to A1 were spectrograms generated by sampling from the hierarchical dynamic model described in the previous section (Figure 2). First, the hidden cause at the 2^nd^ level, i.e. the target log-frequency 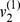, was sampled from a normal distribution; for standards this distribution was centred on the standard frequency and for deviants it was centred on the standard frequency plus the deviance magnitude. Then the sensory input (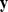) was generated by integrating the HDM's random differential equations with 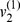 equal to the sampled target frequency. All simulated sensory inputs were generated with low levels of noise, i.e. the precisions were set to 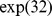. The subject's probabilistic expectation was modelled by a Gaussian prior on the target log-frequency 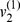. Perception was simulated with generalised filtering of the ensuing sensory input. The generative model of the subject was identical to the model used to generate the inputs, except for the prior belief about the target frequency. The prior belief about the target frequency models prior expectations established by the preceding events, where the mean was set to the standard frequency – and its precision was set according to the deviant probability of the simulated oddball experiment: see Text S1. The noise precisions were chosen to reflect epistemic uncertainty about the process generating the sensory inputs: see Text S1. Note that since we are dealing with dynamic generative models, the prior belief is not just about the initial value, but about the entire trajectory of the target frequency.

**Figure 3:**
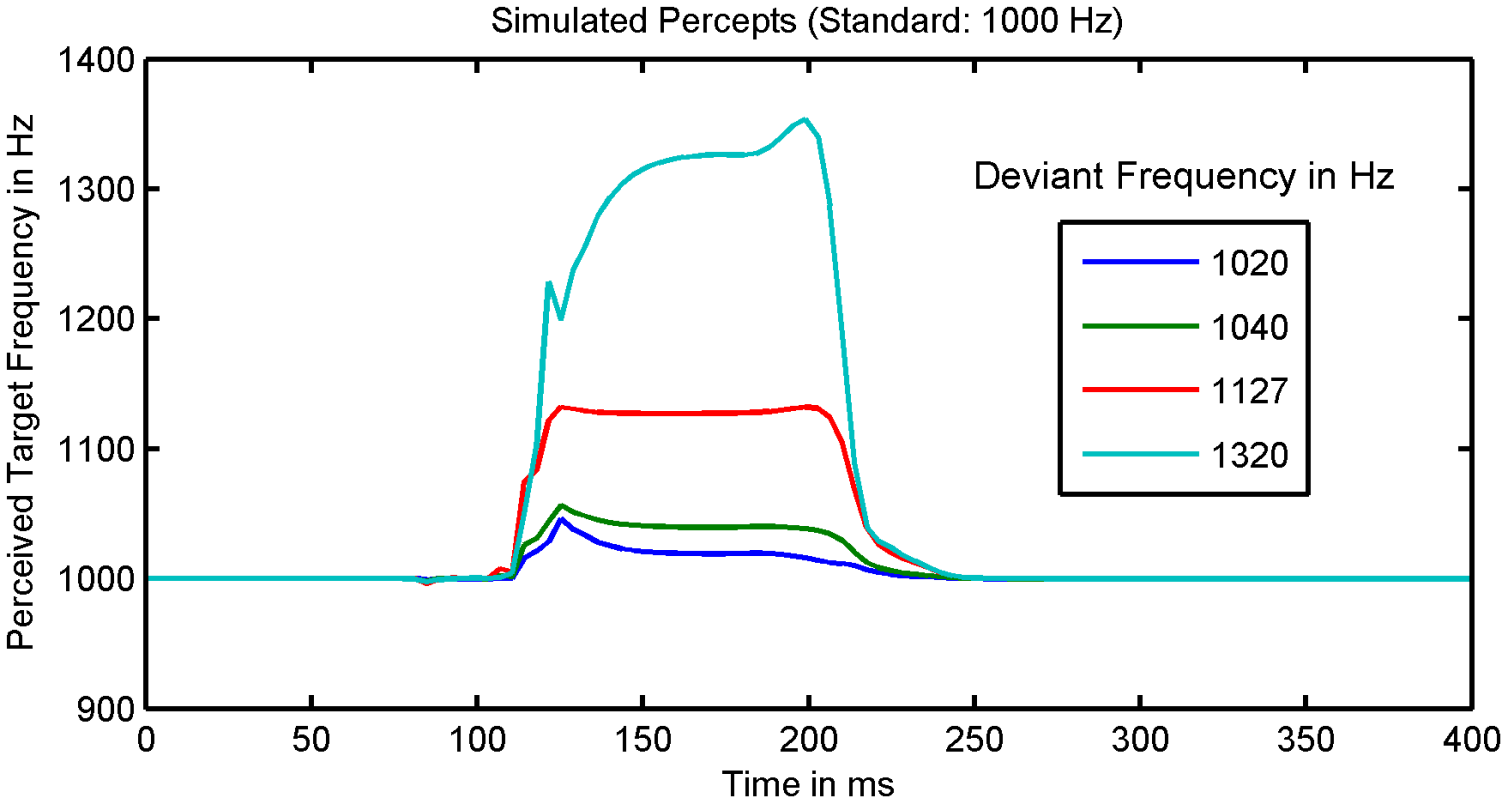
Simulation of perceptual inference. This figure shows the simulated time course of the perceived frequency for four different deviants. The expected frequency was 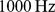 and the frequency of the simulated deviant varied between 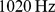 and 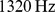. The simulated auditory responses correctly inferred the deviant frequency, despite its discrepancy with its prior expectation. The prior certainty was chosen to correspond to a deviant probability of 0.05.

Figure 3 shows an example of stimulus generation and recognition. This figure shows that the predictive coding scheme correctly inferred the frequency of the tone. In these simulations, the loudness of the stimulus was modulated by a Gaussian bump function that peaks at about 70 ms and has a standard deviation of about 30 ms. The sensory evidence is therefore only transient, whereas prior beliefs are in place before, during, and after sensory evidence is available. As a consequence, the inferred target frequency drops back to the prior mean, when sensory input ceases. Although we are now in a position to simulate neuronal responses to standard and oddball stimuli, we still have to complete the model of observed electromagnetic responses:

### From prediction errors to ERPs

The production of the MMN from prediction errors was modelled as a two stage process: the generation of scalp potentials from neuronal responses and subsequent data processing (see Figure 1). We modelled the scalp potentials (at one fronto-central electrode) as the linear superposition of electromagnetic fields caused by the activity of prediction error units in the three simulated cortical sources – plus background activity. Specifically, prediction error units in the A1 source are assumed to encode 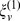 – the precision weighted sensory error; error units in lateral Heschl's gyrus were assumed to encode 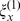 – the precision weighted errors in the motion of hidden (log-frequency and amplitude) states; and prediction error units in the inferior frontal gyrus were assumed to encode 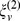 – the precision weighted errors in their inferred causes. The prediction errors were transformed into event related potentials by three transformations. First, the time axis was shifted (to accommodate conduction delays from the ear) and scaled so that the simulated stimulus duration was 70 ms. Second, a sigmoidal transformation was applied to capture the presumably non-linear mapping from signed precision-weighted prediction error to neural activity (i.e. the firing rate cannot be negative and saturates for high prediction error) and in the mapping from neuronal activity to equivalent current dipole activity; these first two steps are summarized by
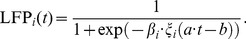
(12)Finally, the scalp potential 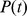 is simulated with a linear combination of the three local field potentials 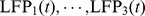 plus a constant:
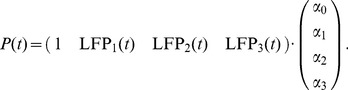
(13)Data processing was simulated by the application of down-sampling to 200 Hz and a 3^rd^ order Butterworth low-pass filtering with a cut-off frequency of 40 Hz, cf. [6], [23], [28], [39]. We performed two simulations for each condition. In the first simulation the subject expected stimulus A but was presented with stimulus B (deviant). In the second simulation, the subject expected stimulus B and was presented with stimulus B (standard). The MMN was estimated by the difference wave (deviant ERP – standard ERP). This procedure reproduces the analysis used in electrophysiology [7], [40].

This completes the specification of our computationally informed dynamic causal model of the MMN.

To explore the predictions of this model under different levels of deviant probability and magnitude, we first estimated the biophysical parameters (i.e. the slope parameters 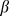 in (12) and the lead field 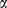 in (13)) from the empirical ERPs described in [19], using standard nonlinear least-squares techniques (i.e. the GlobalSearch algorithm [41] from the Matlab Global Optimization toolbox). We then used the estimated parameters to predict the MMN under different combinations of deviant probability and magnitude.

In particular, the simulated MMN waveforms were used to reproduce the descriptive statistics typically reported in MMN experiments, i.e. MMN amplitude and latency. MMN latency was estimated by the fractional area technique [19], because it is regarded as one of the most robust methods for measuring ERP latencies [42]. Specifically, we estimated the MMN latency as the time point at which 50% of the area of the MMN trough lies on either side. This analysis was performed on the difference wave between the first and last point at which the amplitude was at least half the MMN amplitude. This analysis was performed on the unfiltered MMN waveforms as recommended by [43]. MMN amplitude was estimated by the average voltage of the low-pass filtered MMN difference wave within a ±10 ms window around the estimated latency.

## Results

### Simulated ERPs

**Figure 4:**
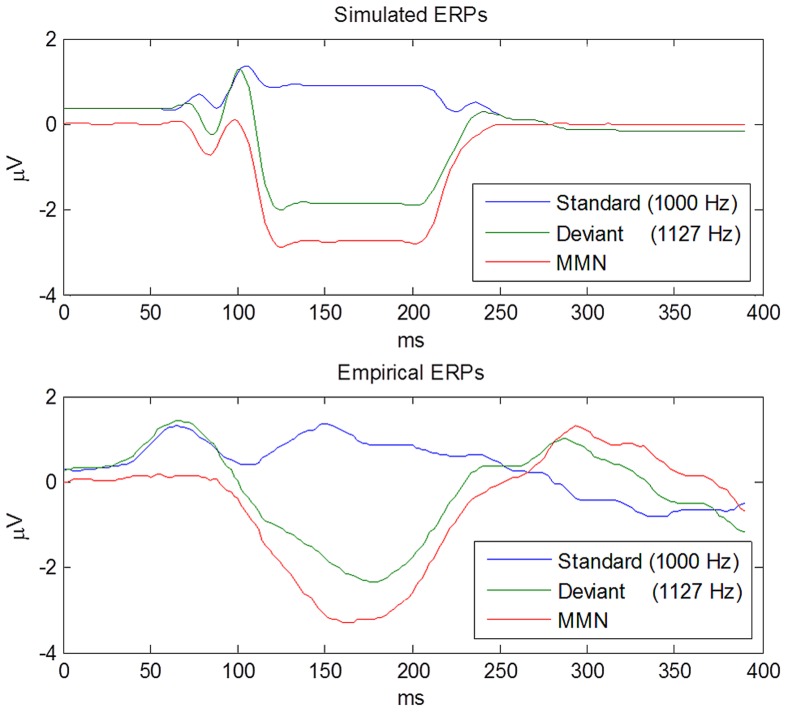
Simulated ERPs vs. empirical ERPs. This figure compares the simulated ERPs evoked by the standard and the deviant, and their difference – the MMN – to the empirical ERPs from [70], [71] to which the model was fitted. The simulation captures both the positivity evoked by the standard and the negativity evoked by the deviant.

Figure 4 shows that the waveforms generated by our model reproduce the characteristic shape of the MMN, the positivity evoked by the standard and the negativity evoked by the deviant. The latency of the simulated MMN (164 ms) was almost identical to the latency of the empirical MMN (166 ms). Its peak amplitude (−2.71 µV) was slightly higher than for the empirically measured MMN (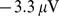), and its width at half-maximum amplitude (106 ms) was also very similar to the width of the empirical MMN waveform (96 ms). In short, having optimised the parameters mapping from the simulated neuronal activity to empirically observed responses, we were able to reproduce empirical MMNs remarkably accurately. This is nontrivial because the underlying neuronal dynamics are effectively solving a very difficult Bayesian model inversion or filtering problem. Using these optimised parameters, we proceeded to quantify how the MMN waveform would change with deviance magnitude and probability.

To simulate the effect of deviant probability, we simulated the responses to a deviant under different degrees of prior certainty. To simulate the effect of deviance magnitude, we varied the discrepancy between the expected and observed frequency, while keeping the deviant probability constant. Finally, we investigated potential interactions between deviance magnitude and deviant probability by simulating the effect of magnitude under different prior certainties and *vice versa*.

#### Qualitative comparisons to empirical data

**Figure 5:**
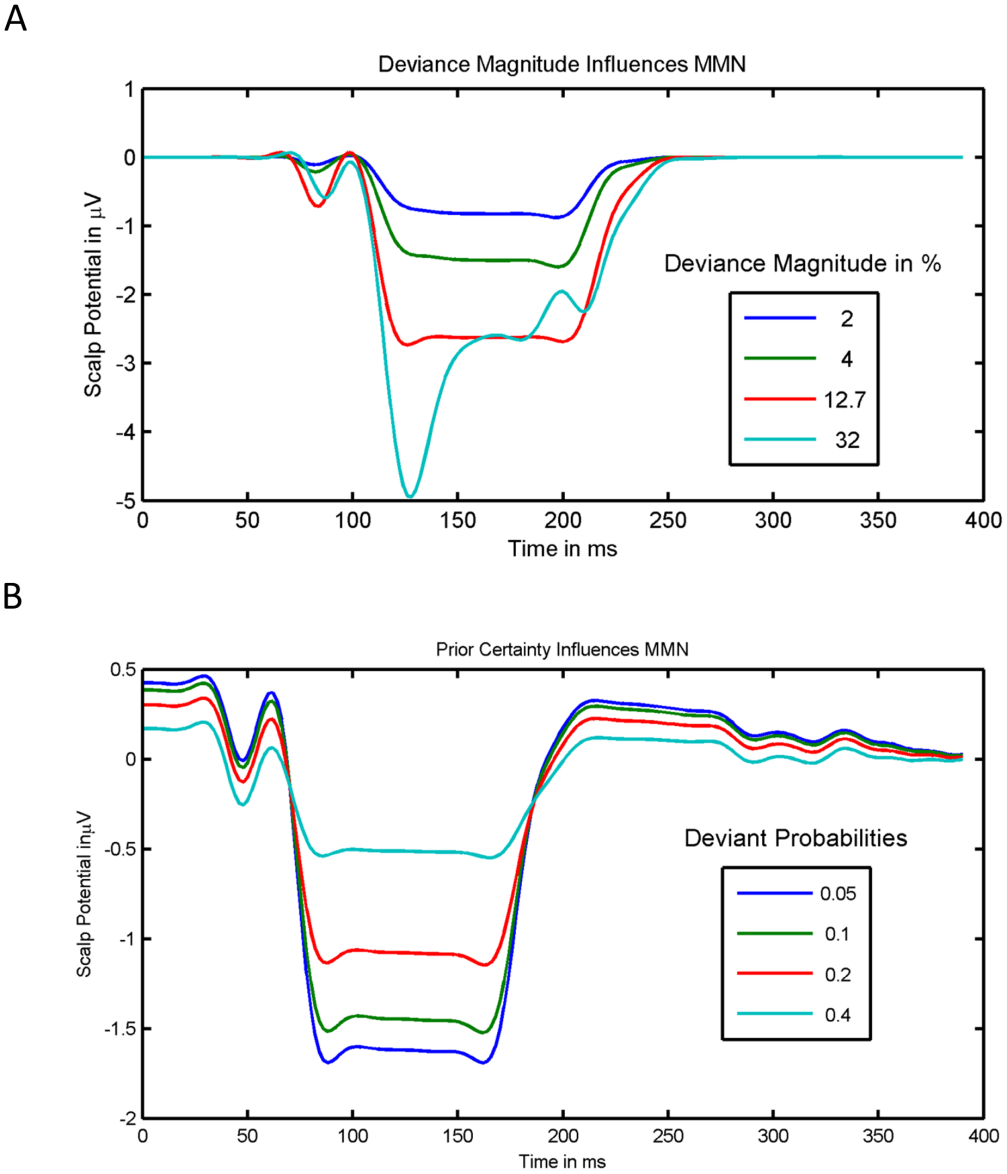
Simulated effects of deviance magnitude and deviant probability. This figure shows the simulated effect of deviance magnitude (panel A) and deviant probability (panel B) on the MMN waveform. As the deviance magnitude increases, the trough becomes deeper and wider and an early peak emerges (panel A). As deviant probability is decreased, the depth of the MMN's trough increases, whereas its latency does not change (panel B). In panel A, the standard frequency was 1000 Hz, the corresponding deviance frequencies were 1020 Hz, 1040 Hz, 1270 Hz, and 1320 Hz, and the simulated deviant probability was 0.05. In panel B, the deviance magnitude was 12.7% (standard: 1000 Hz, deviant 1270 Hz).

**Figure 6:**
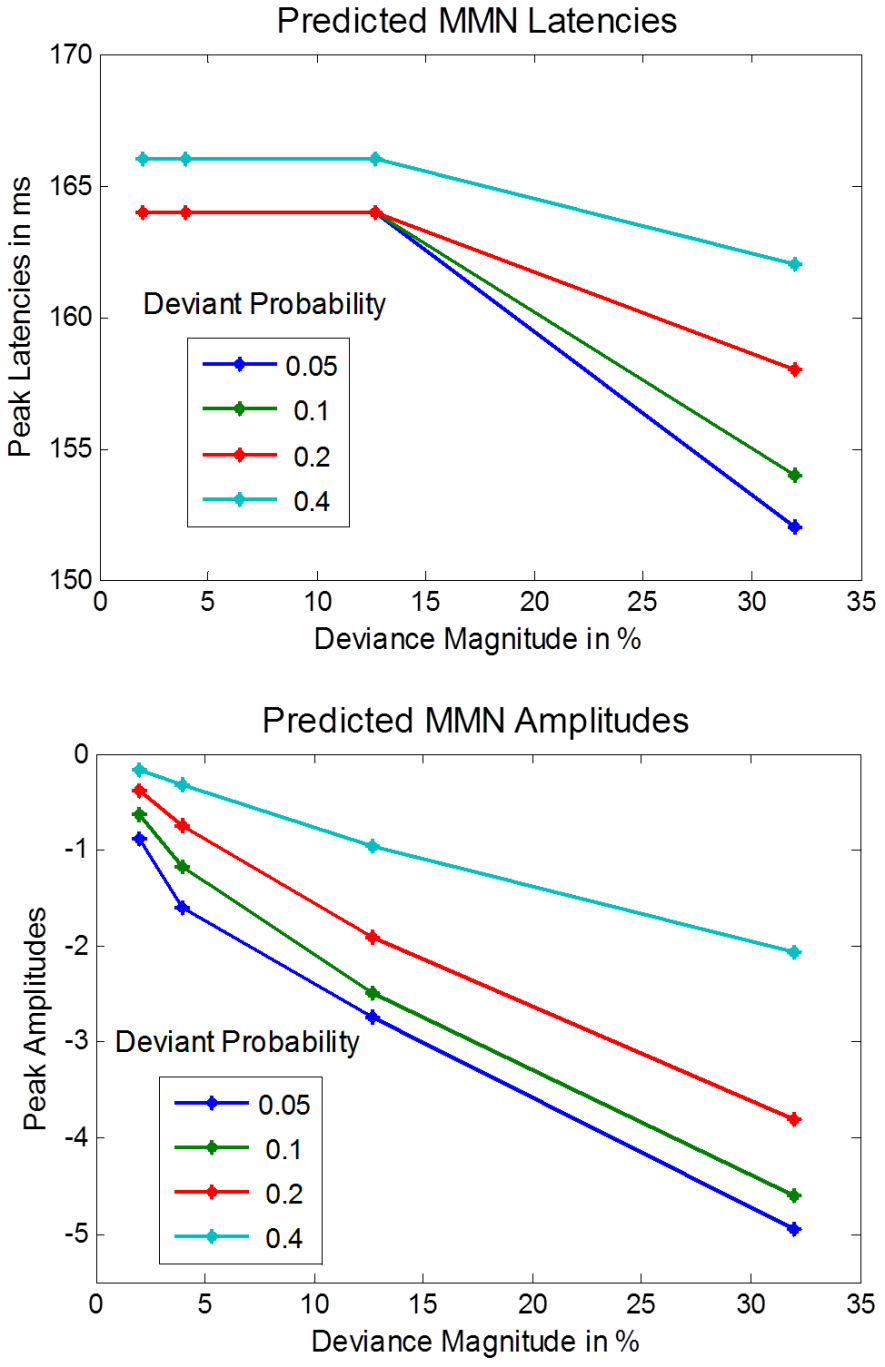
Simulated MMN phenomenology. Our simulations predict that deviance magnitude increases the MMN peak amplitude and shortens its latency. Furthermore, our simulations suggest that when the deviant probability is decreased, the peak amplitude increases, while its latency does not change. The deviance magnitude is specified relative to the standard frequency of 1000

To establish the model's face validity, we asked whether it could replicate the empirically established qualitative effects of deviant probability and magnitude summarized in Table 1. Figure 5a shows the simulated effects of deviance magnitude on the MMN for a deviant probability of 0.05. As the deviance magnitude increases from 2% to 32% the MMN trough deepens. Interestingly, this deepening is not uniform across peristimulus time, but it is more pronounced at the beginning. In effect, the shape of the MMN changes, such that an early peak emerges and the MMN latency decreases. The effects of deviance magnitude on MMN peak amplitude and latency hold irrespective of the deviant probability: see Figure 6. In short, our model correctly predicts the empirical effects of deviance magnitude on MMN amplitude and latency (Table 1).

Figure 5b shows the effect of deviant probability on the MMN for a deviance magnitude of 12.7%. As the probability of a deviant decreases, the MMN trough deepens, but its shape and centre remain unchanged. As with empirical findings (Table 1), our simulations suggest that the amplitude of the MMN's peak increases with decreasing deviant probability, but its latency is unaffected. Figure 6 summarizes the peak amplitudes and latencies of the simulated MMN as a function of deviance magnitude and probability. As the upper plot shows, the MMN peak amplitude increases with deviance magnitude and decreases with deviant probability. Furthermore, deviance magnitude appears to amplify the effect of deviant probability and vice versa. The lower plot shows that the MMN latency is shorter when deviance magnitude is 32% than when it is 12.7%. These results also suggest that the deviant probability has no systematic effect on MMN latency – if the deviance magnitude is at most 12.7% and deviant probability is below 40%. However, they predict that MMN latency shortens with decreasing deviant probability – if deviance magnitude is increased to 32% or deviant probability is increased to 40%.

Furthermore, our model predicts that MMN amplitude is higher when the deviant is embedded in a stream of standards (deviant condition) than when the same tone is embedded in a random sequence of equiprobable tones (control condition) [44], [45]: In the control condition – with its equiprobable tones – the trial-wise prediction about the target frequency is necessarily less precise. As a result, the neural activity encoding the precision weighted prediction error about the target frequency will be lower, so that the deviant negativity will be reduced relative to the deviant condition. This phenomenon cannot be explained by the spike-frequency adaptation in narrow frequency channels [44], but see [46]-[50] for a demonstration that it can be explained by synaptic depression.

#### Quantitative comparisons to empirical data

**Figure 7:**
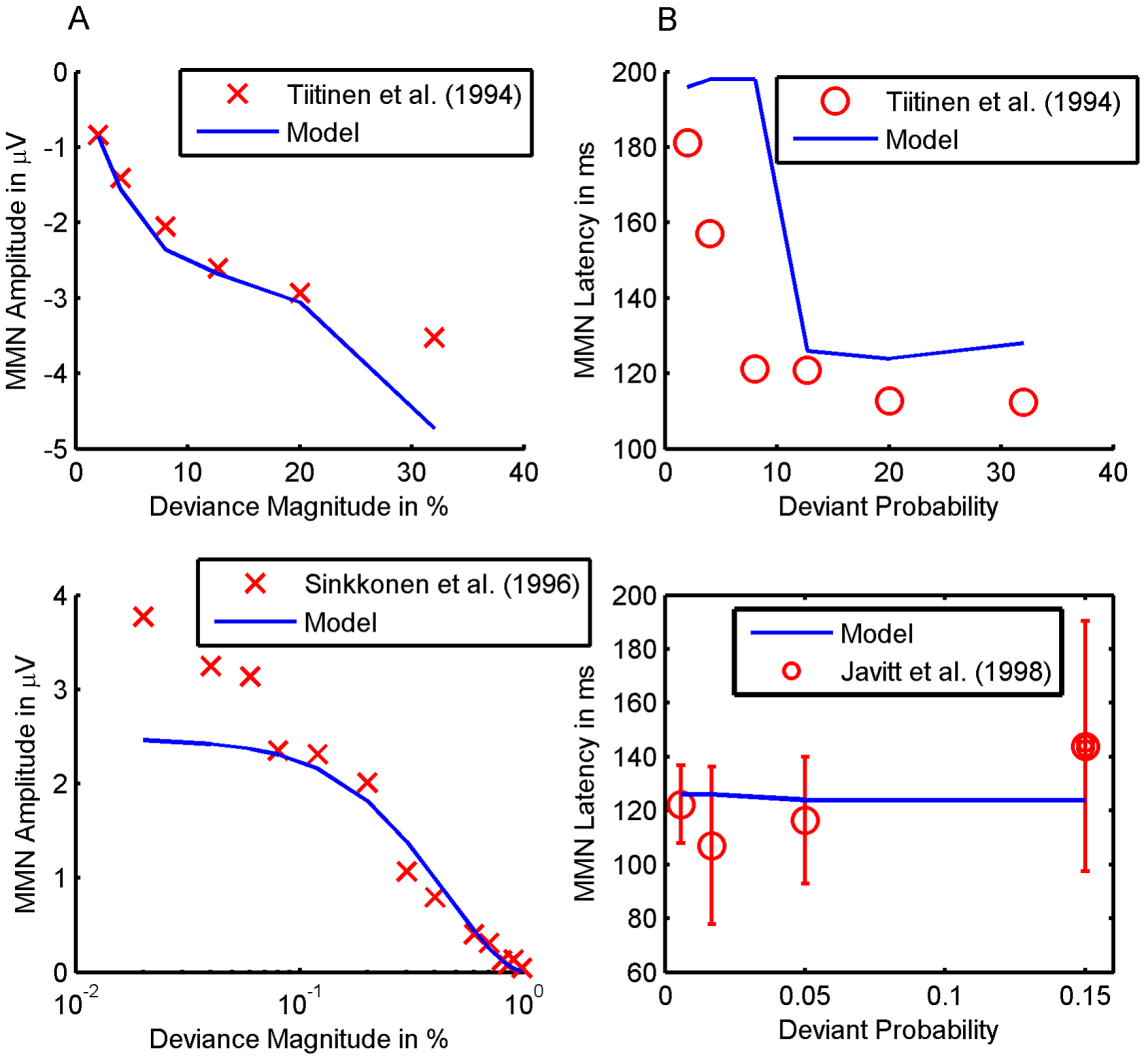
Quantitative model fit of MMN amplitude and latency. This figure compares predictions about the MMN amplitude (panel A) and latency (panel B) with empirical data from auditory oddball experiments. The upper plot in panel A is based on [72], where deviance magnitude was varied for a fixed deviant probability of 0.05. The lower plot in panel A is based on [19], where deviant probability was varied for a fixed deviance magnitude of 15% (deviant frequency: 1150 Hz, standard frequency: 1000 Hz). The upper plot in panel B is based on the same experiment [9] as the upper plot in panel A. The lower plot in panel B is based on [10], where deviant probability was varied with a fixed deviance magnitude of 20% (deviant frequency 1200 Hz, standard frequency 1000 Hz). The error bars indicate the standard error of the mean.

Having established that the model reproduces the effects of deviant probability and magnitude on MMN amplitude and latency in a qualitative sense, we went one step further and assessed quantitative predictions. For this purpose, we simulated three MMN experiments and reproduced the analyses reported in the corresponding empirical studies. We found that the effects of deviance magnitude and probability on the MMN peak amplitude matched the empirical data of [51] and [52] not only qualitatively but also quantitatively (see Figure 7a). Our model explained 93.6% of the variance due to deviance magnitude reported in [9] (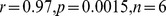) and 93.2% of the variance due to deviant probability reported in [10] (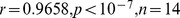). Furthermore, we simulated two experiments that investigated how the MMN latency depends on deviance magnitude [9] and probability [10] (see Figure 7b). The model correctly predicted the absence of an effect of deviant probability on MMN latency in a study where the deviance magnitude was 20% [9]. While our model predicted that the MMN latency is shorter for high deviance magnitudes than for low deviance magnitudes, it also predicted a sharp transition between long MMN latencies (195 ms) for deviance magnitudes up to 12.7% and a substantially shorter MMN latency (125 ms) for a deviance magnitude of 32%. By contrast, the results reported in [11] appear to suggest a gradual transition between long and short MMN latencies. In effect, the model's predictions explained only 51.9% of the variance of MMN latency as a function of deviance magnitude [11] (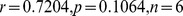).

## Discussion

We have described a process model of the MMN and its dependence on deviant stimulus (deviance magnitude) and context (deviant probability). Together with the study presented in [9], this work demonstrates the potential of predictive coding to provide a comprehensive explanation of MMN phenomenology. More precisely, our model explains the effects of deviant probability and magnitude on the MMN amplitude under the assumption that evoked responses reflect the neuronal encoding of (precision weighted) prediction errors. The simulated MMN was a superposition of the electrical fields generated by prediction errors at different hierarchical levels of representation (see Figure 2), where their relative contributions (i.e. the coefficients in equation (13)) differed: the errors in the predictions at the highest level of representation (inferior frontal gyrus) were weighted most strongly, followed by prediction error at the sensory level (A1) and prediction errors at the intermediate level (lateral Heschl's gyrus). As a result, the simulated MMN primarily reflected prediction errors on the hidden causes (attributes), rather than prediction errors on their physical features.

Our model offers a simple explanation as to why the MMN amplitude decreases with deviant probability and increases with deviance magnitude. Precision weighted prediction errors are the product of a prediction error and the precision of the top-down prediction. Hence, according to our model, deviance magnitude increases MMN amplitude, because it increases prediction errors. Similarly decreasing the probability of the deviant increases the MMN amplitude by increasing the precision of (learned) top-down predictions. Furthermore, since precision and prediction error interact multiplicatively, the precision determines the gain of the effect of prediction error and *vice versa*.

This model explains the shortening of the MMN latency with deviance magnitude by a selective amplification of frequency-related prediction errors that are only transiently expressed – because they are explained away quickly by top-down predictions. These prediction errors increase with deviance magnitude. However, there are also prediction errors that are not explained away by perceptual inference. These errors are sustained throughout the duration of the stimulus (as the stimulus amplitude fluctuates) and do not depend on the difference between the standard and the deviant event. Hence, according to our model, deviance magnitude selectively increases the early prediction error component, but not sustained errors. In effect, as deviance magnitude increases, an early trough emerges within the MMN, so that the MMN latency shortens (see Figure 5a and Figure 6). By contrast, increasing the precision of high-level beliefs increases all precision weighted frequency prediction errors – the transient and the sustained – equally. Thus the MMN deepens uniformly, and no early trough emerges. This is why – according to the model – the deviant probability has no effect on the MMN latency for moderate deviance magnitudes. However, if the deviance magnitude is so large that the transient component dominates the frequency-related prediction error, the situation is different. In this case, increasing the weight of the frequency-related prediction errors relative to loudness-related prediction errors can shorten the latency, because the frequency-related prediction error predominates at the beginning of perception – whereas the amplitude related prediction error is constant throughout perception. This is why our model predicts that the MMN latency becomes dependent on deviant probability at higher levels of deviance magnitude.

### Novel predictions

Our MMN simulations predict a nonlinear interaction between the effects of deviant probability and magnitude. The upper plot in Figure 6 suggests that the effect of deviant probability on MMN peak amplitude increases with increasing deviance magnitude. Conversely, the effect of deviance magnitude increases with decreasing deviant probability. Furthermore, the lower plot in Figure 6 suggests, that the effect of deviant probability on MMN latency depends on deviance magnitude: If deviance magnitude is at most 12.7%, the MMN latency does not depend on deviant probability, but when deviance magnitude is as large as 32%, the MMN latency increases with deviant probability. Conversely, the size of the effect of deviance magnitude on MMN latency depends on deviant probability. Hence, our simulations predict a number of interaction effects that can be tested empirically.

### Relation to previous work

Although the physiological mechanisms generating the MMN have been modelled previously [9], the model presented here is the first to bridge the gap between the computations implicit in perceptual inference and the neurophysiology of ERP waveforms. In terms of Marr's levels of analysis [53], our model provides an explanation at both the algorithmic and implementational levels of analysis – and represents a step towards full meta-Bayesian inference – namely inferring from measurements of brain activity on how the brain computes (cf. [13], [19], [51]–[55]).

Our model builds upon the proposal that the brain inverts hierarchical dynamic models of its sensory inputs by minimizing free-energy in a hierarchy of predictive coding circuits [56]. Specifically, we asked whether the computational principles proposed in [15], [20] are sufficient to generate realistic MMN waveforms and account for their dependence on deviant probability and deviance magnitude. In doing so, we have provided a more realistic account of the algorithmic nature of the brain's implementation of these computational principles: While previous simulations have explored the dynamics of perceptual inference prescribed by the free-energy principle using dynamic expectation maximization (DEM) [23], [39], the simulations presented here are based on GF [23], [39]. Arguably, GF provides a more realistic model of learning and inference in the brain than DEM, because it is an online algorithm that can be run in real-time to simultaneously infer hidden states and learn the model; i.e., as sensory inputs arrive. In contrast to DEM it does not have to iterate between inferring hidden states, learning parameters, and learning hyperparameters. This is possible, because GF dispenses the mean-field assumption made by DEM. Another difference to previous work is that we have modelled the neural representation of precision weighted prediction error by sigmoidal activation functions, whereas previous simulations ignored potential nonlinear effects by assuming that the activity of prediction error units is a linear function of precision weighted prediction error [6], [24], [27], [39]. Most importantly, the model presented here connects the theory of free-energy minimisation and predictive coding to empirical measurements of the MMN in human subjects.

To our knowledge, our model is the first to provide a computational explanation of the MMN's dependence on deviance magnitude, deviant probability, and their interaction. While [26] modelled the effect of deviance magnitude, they did not consider the effect of deviant probability. Although [6], [24] modelled the effect of deviant probability, they did not simulate the effect of deviance magnitude, nor did they make quantitative predictions of MMN latency or amplitude. Mill et al. [13], [55] simulated the effects of deviance magnitude and deviant probability on the firing rate of single auditory neurons in anaesthetized rats. While their simulations captured the qualitative effects of deviance magnitude and deviant probability on response amplitude, they did not capture the shortening of the MMN latency with decreasing deviant probability. By contrast, our model generates realistic MMN *waveforms* and explains the qualitative effects of deviant probability and magnitude on the amplitude and latency of the MMN. Beyond this, our model makes remarkably accurate quantitative predictions of the MMN amplitude across two experiments [53] examining several combinations of deviance magnitude and deviant probability.

### Limitations

The simulations reported in this paper demonstrate that predictive coding can explain the MMN and certain aspects of its dependence on the deviant stimulus and its context. However, they do not imply that the assumptions of predictive coding are necessary to explain the MMN. Instead, the simulations are a proof-of-concept that it is possible to relate the MMN to a process model of how prediction errors are encoded dynamically by superficial pyramidal cells during perceptual inference. For parsimony, our model includes only those three intermediate levels of the auditory hierarchy that are assumed to be the primary sources of the MMN. In particular, we do not model the subcortical levels of the auditory system. However, our model does *not* assume that predictive coding starts in primary auditory cortex. To the contrary, the input to A1 is assumed to be the prediction error from auditory thalamus. This is consistent with the recent discovery of subcortical precursors of the MMN [52]. Since MMN waveforms were simulated using the parameters estimated from the average ERPs reported in [9], [10], the waveforms shown in Figure 4 are merely a demonstration that our model can fit empirical data. However, the model's ability to predict how the MMN waveform changes as a function of deviance magnitude and deviant probability speaks to its face validity.

Our model's most severe failure was that while our model correctly predicted that MMN latency shortens with deviance magnitude, it failed to predict that this shortening occurs gradually for deviance magnitudes between 2.5% and 7.5%. In principle, the model predicts that the latency shortens gradually within a certain range of deviance magnitudes, but this range did not coincide with the one observed empirically.

There are clearly many explanations for this failure – for example, an inappropriate generative model or incorrect forms for the mapping between prediction errors and local field potentials. Perhaps the more important point here is that these failures generally represent opportunities. This is because one can revise or extend the model and compare the evidence for an alternative model with the evidence for the original model using Bayesian model comparison of dynamic causal models in the usual way [57]–[59]. Indeed, this is one of the primary motivations for developing dynamic causal models that are computationally informed or constrained. In other words, one can test competing hypotheses or models about both the computational (and biophysical) processes underlying observed brain responses.

### Conclusions

This work is a proof-of-principle that important aspects of evoked responses in general – and the MMN in particular – can be explained by formal (Bayesian) models of the predictive coding mechanism [19]. Our model explains the dynamics of the MMN in continuous time and some of its phenomenology at a precision level that has not been attempted before. By placing normative models of computation within the framework of dynamic causal models one has the opportunity to use Bayesian model comparison to adjudicate between competing computational theories. Future studies might compare predictive coding to competing accounts such as the fresh-afferent theory [60]–[62]. In addition, the approach presented here could be extended to a range of potentials evoked by sensory stimuli, including the N1 and the P300, in order to generalise the explanatory scope of predictive coding or free energy formulations.

This sort of modelling approach might be used to infer how perceptual inference changes with learning, attention, and context. This is an attractive prospect, given that the MMN is elicited not only in simple oddball paradigms, but also in more complex paradigms involving the processing of speech, language, music, and abstract features [7], [53], [63]. Furthermore, a computational anatomy of the MMN might be useful for probing disturbances of perceptual inference and learning in psychiatric conditions, such as schizophrenia [13], [55]. Similarly, extensions of this model could also be used to better understand the effects of drugs, such as ketamine [12], [64]–[66], or neuromodulators, such as acetylcholine [67]–[69], on the MMN. We hope to pursue this avenue of research in future work.

## Supporting Information

Text S1 **Modelling assumptions about tuning curves in primary auditory cortex and the brain's prior uncertainty.** The supplementary text details and justifies our model's assumptions about the tuning curves of neurons in primary auditory cortex and the covariance matrices in the perceptual model.
(DOCX)
