# Correction: A Neurocomputational Model of the Mismatch Negativity

**DOI:** 10.1371/annotation/ca4c3cdf-9573-4a93-9542-3a62cdbb8396

**Published:** 2013-12-11

**Authors:** Falk Lieder, Klaas E. Stephan, Jean Daunizeau, Marta I. Garrido, Karl J. Friston

Beginning at “with physiologically grounded models of neuroimaging data…” in the first paragraph of the Introduction, the citations are incorrect throughout the text.

The correct citation(s) for each citation block, delineated by [X], can be found two blocks further on in the text. 

For example: in the Introduction section, the sentence “…with physiologically grounded models of neuroimaging data [4,5]” should instead cite reference [1] from the paragraph below.

Consequently the last line of the manuscript is incorrect and should read:

“Similarly, extensions of this model could also be used to better understand the effects of drugs, such as ketamine [70,71], or neuromodulators, such as acetylcholine [72], on the MMN. We hope to pursue this avenue of research in future work.” 

