# Supplementary material for: A Neurocomputational Model of the Mismatch Negativity
Source: PLoS Comput Biol. 2013 Nov 7;9(11):e1003288. doi: 10.1371/journal.pcbi.1003288 (PMC3820518; doi:10.1371/journal.pcbi.1003288)
Supplement: Text S1 — Modelling assumptions about tuning curves in primary auditory cortex and the brain's prior uncertainty. The supplementary text details and justifies our model's assumptions about the tuning curves of neurons in primary auditory cortex and the covariance matrices in the perceptual model. (DOCX) [file pcbi.1003288.s001.docx]

Text S1

## A1 Tuning Curves

In the main text, the response of A1 neurons with a preferred frequency $\omega_{i}$ and specificity determined by $\sigma$ is defined by

Here, we describe how $\sigma$ and the preferred frequencies $\omega_{i}$ were chosen. In auditory neurophysiology, a neuron’s tuning curve is typically characterized by how loud a tone with a given frequency has to be to fire the neuron. A substantial fraction of neurons appears to have a V-shaped tuning curve. Empirically, the tone has to be twice as loud when its frequency is 7/8 or 9/8 of the neuron’s best frequency [[1](#_ENREF_1)]. We have chosen $\sigma^{2}$ of the tuning curve of A1 neurons such that the simulated responses comply with this observation:

$\mathcal{N}\left( \omega_{i};\omega_{i},\sigma^{2} \right)=2\cdot\mathcal{N}\left( \omega_{i}+\log\left( \frac{9}{8} \right);\omega_{i},\sigma^{2} \right)$

implies $\sigma^{2}=\frac{{\log(9/8)}^{2}}{2\cdot\log(2)}\approx0.01$ and $\mathcal{N}\left( \omega_{i};\omega_{i},\sigma^{2} \right)=2\cdot\mathcal{N}\left( \omega_{i}+\log\left( \frac{7}{8} \right);\omega_{i},\sigma^{2} \right)$

implies $\sigma^{2}\approx0.013$

We therefore set $\sigma^{2}$ to 0.0115. We model 50 (A1) frequency channels whose preferred log-frequencies are equidistantly distributed between $\log(900 \mathrm{Hz})$ and $\log(1400 \mathrm{Hz})$ – to ensure a sufficient coverage of the frequencies used in the simulated experiments (1000 Hz-1320 Hz).

## Prior Certainties

The proposed generative model (Figure 2) captures three kinds of uncertainty. The first is the uncertainty about the spectrogram representation in primary auditory cortex and is captured by $\iota_{y}$, the log-precision of the observation noise:

$$\text{Σ}_{y}=\exp\left(-\iota_{y}\right)\cdot\left(\begin{matrix} 1 & 0 & \cdots\\ 0 & \ddots& 0 \\ \vdots& 0 & 1 \end{matrix}\right).$$

Second, there may be uncertainty about how the trajectory of frequency and loudness changes that is captured by $\iota_{x}$, the log precision of the state noise:

$$\text{Σ}_{x}=\exp\left(-\iota_{x}\right)\cdot\left(\begin{matrix} 1 & 0 & \cdots\\ 0 & \ddots& 0 \\ \vdots& 0 & 1 \end{matrix}\right).$$

finally, there is uncertainty about what the frequency and the loudness underlying these changes. This is captured by $\iota_{v}$, the log precision of the fluctuations about the cause:

$$\text{Σ}_{v}=\exp\left(-\iota_{v}\right)\cdot\left(\begin{matrix} 1 & 0 & \cdots\\ 0 & \ddots& 0 \\ \vdots& 0 & 1 \end{matrix}\right)$$

These three precisions were chosen according to the following considerations:

1. The log precision of the observation noise was chosen such that the signal to noise ratio (SNR) of the simulated sound matches the SNR of a typical auditory oddball experiment. Specifically, we assumed that the background noise in the laboratory is about 20 dB, whereas the loudness of the tones is 80 dB. This corresponds to a SNR of 60 dB. This means that $10\cdot\log_{10} \left( \frac{\left\| S \right\|_{2}^{2}}{\left\| N \right\|_{2}^{2}} \right)=60$ and therefore $\left\| N \right\|_{2}^{2}={10}^{-6}\cdot\left\| S \right\|_{2}^{2} MACROBUTTON MTPlaceRef \backslash* MERGEFORMAT SEQ MTEqn \backslash h \backslash* MERGEFORMAT ( SEQ MTEqn \backslash c \backslash* Arabic \backslash* MERGEFORMAT 9)$. The log power of the simulated signal was about 0.0366 units per frequency channel. This implies that the power of the noise should be about $3.66\cdot{10}^{-8}$ units per frequency channel. Therefore the log noise precision should be

$\iota_{y}=-\ln\left(3.66\cdot10^{-8}\right)=8\ln\left(10\right)-\ln\left(3.66\right)\approx17.12$,

which results in a signal-to-noise ratio of 60 dB.

1. The change in frequency and loudness was assumed to be a deterministic function of the true frequency and the true loudness respectively, i.e. $\iota_{x}=32 MACROBUTTON MTPlaceRef \backslash* MERGEFORMAT SEQ MTEqn \backslash h \backslash* MERGEFORMAT ( SEQ MTEqn \backslash c \backslash* Arabic \backslash* MERGEFORMAT 11)$.
2. The precision of the prior belief about the cause of the temporal evolution of the log-frequency was chosen such that the subjective certainty that the standard event will occur is 1 minus the deviant probability $p$. The standard event was defined by the occurrence of a frequency that differs from the standard frequency by less than half of the half-tone step of a musical scale. Hence, since there are 12 half-tone steps in each octave and the frequency doubles within each octave, the difference in log-frequency has to be below log(2)/24 in order for the tone to be considered the standard. Based on this definition, prior certainties were chosen to express the beliefs that the deviant will be presented with probability $p$ of 0.025, 0.05, 0.1, 0.2, and 0.4, according to . $\begin{matrix} \text{P}\left(\log(f_{\text{std}})-\text{log}(2)/24\leq v_{0}^{\left(2\right)}\leq\log\left(f_{\text{std}}\right)+\text{log}(2)/24);\mu=\log(f_{\text{std}}),\sigma_{v^{(2)}}\right)=1-p \\ \Rightarrow1-p=1-2\cdot\text{Φ}(-\log\left(2\right)/(24\text{σ}_{v^{(2)}})) \\ \Rightarrow\sigma_{v^{(2)}}=-\frac{\log\left(2\right)}{24\text{Φ}^{-1}\left(p/2\right)}\Rightarrow\iota_{v_{2}}=\left(\frac{24\text{Φ}^{-1}\left(p/2\right)}{\log\left(2\right)}\right)^{2} \end{matrix}$
3. The prior belief about the cause of the temporal evolution of loudness was chosen to reflect perfect knowledge of its trajectory. The prior mean was set to the true mean and the log prior certainty was set to ${{\iota_{v_{1}}}}=32$.

# References

1. Sadagopan S, Wang X (2008) Level Invariant Representation of Sounds by Populations of Neurons in Primary Auditory Cortex. The Journal of Neuroscience 28: 3415-3426.
